# The Association between Treatment for Metabolic Disorders and Breast Cancer Characteristics

**DOI:** 10.1155/2016/4658469

**Published:** 2016-08-28

**Authors:** Hadar Goldvaser, Shulamith Rizel, Daniel Hendler, Victoria Neiman, Daniel Shepshelovich, Tzippy Shochat, Aaron Sulkes, Baruch Brenner, Rinat Yerushalmi

**Affiliations:** ^1^Institute of Oncology, Davidoff Cancer Center, Beilinson Hospital, Rabin Medical Center, 39 Jabotinski St., Petah Tikva, Israel; ^2^Sackler Faculty of Medicine, Tel Aviv University, P.O. Box 39040, Tel Aviv, Israel; ^3^Department of Medicine A, Beilinson Hospital, Rabin Medical Center, 39 Jabotinski St., Petah Tikva, Israel; ^4^Statistical Consulting Unit, Beilinson Hospital, Rabin Medical Center, 39 Jabotinski St., Petah Tikva, Israel

## Abstract

*Purpose*. To evaluate the associations between metformin, insulin, statins, and levothyroxine and breast cancer characteristics and outcome.* Methods*. Retrospective chart review of patients treated in our institute for early estrogen receptor (ER) positive, human epidermal growth factor receptor 2 negative breast cancer, whose tumors were sent to Oncotype DX (ODX) analysis. Patients were grouped according to medications usage during the time of breast cancer diagnosis. Each group was compared to the rest of the study population.* Results*. The study cohort included 671 patients. Sixty (9.1%) patients were treated with metformin, 9 (1.4%) with insulin, 208 (31.7%) with statins, and 62 (9.4%) with levothyroxine. Patients treated with metformin had more intense ER stain (*p* = 0.032) and a lower ODX recurrence score (RS) (*p* = 0.035). Diagnosis of diabetes mellitus was also associated with lower ODX RS (*p* = 0.014). Insulin usage was associated with a higher rate of angiolymphatic invasion (*p* = 0.041), but lower Ki67% (*p* = 0.017). Levothyroxine usage was associated with different histological subtype distribution (*p* = 0.02). Extended levothyroxine usage was associated with lower ODX RS (*p* = 0.005). Statin usage had no impact on tumor characteristics. Outcome was comparable in the studied subgroups.* Conclusions*. Common medications for metabolic disorders might be associated with breast cancer characteristics.

## 1. Introduction

Breast cancer is a heterogonous disease. Treatment and outcome are influenced by various parameters, including tumor size, nodal involvement, grade, estrogen receptor (ER), progesterone receptor (PR), and human epidermal growth factor receptor 2 (HER2) statuses, as well as Ki67 percentage and the presence of angiolymphatic invasion [1–8]. All of these parameters are well established prognostic factors for disease recurrence. Modern oncology has added to these parameters the multigene assays, such as Oncotype DX (ODX), which provides additional prognostic and predictive information, based on a real time polymerase chain reaction (RT-PCR) of 21 genes. A mathematical formula which uses the quantified results generates a score that predicts distant relapse and provides information regarding the potential benefit of adjuvant chemotherapy in early ER positive and HER2 negative breast cancer [9, 10].

The associations between different metabolic disorders especially obesity and diabetes mellitus (DM) and breast cancer are well established [11–16]. However, data regarding the impact of drugs commonly used to treat these conditions on breast cancer are scarce.

Metformin is a commonly used, oral antidiabetic agent that decreases hyperinsulinemia. As hyperinsulinemia and insulin-like growth factors were reported to have mitogenic effect on breast cells [17, 18], metformin may favorably affect patients with breast cancer. Many* in vitro* and* in vivo* studies found that metformin inhibits cancer cell growth, reduces the risk of developing solid tumors, and improves cancer outcome, including breast cancer [11, 19–23]. The reduced risk of breast cancer associated with metformin use was clearly demonstrated in a recent large epidemiological study [24]. Furthermore, metformin usage was associated with a threefold greater pathologic complete response in diabetic patients with breast cancer receiving neoadjuvant chemotherapy compared to patients with DM not treated with metformin [25]. In addition, a meta-analysis by Xu et al. found that breast cancer patients with DM who were treated with metformin had significantly decreased risk of all-cause and cancer specific mortality compared with their counterparts who did not receive metformin [26]. In light of these data, the drug is currently being investigated for adjuvant breast cancer treatment of nondiabetic patients (https://clinicaltrials.gov/ct2/show/NCT01101438, NCIC CTG MA.32 study, Goodwin PJ et al.). On the other hand, several reports did not support the positive impact of metformin on breast cancer [21, 27–30].

The activity of insulin as a growth factor has raised concern regarding the stimulation of neoplastic growth by insulin analogues. Data regarding the influence of insulin analogues on cancer incidence are inconsistent. One meta-analysis found that diabetic patients treated with insulin had an increased cancer risk compared to non-insulin-treated diabetics [31], while another study reported an inconsistent impact of insulin on cancer incidence [32]. More specifically, a meta-analysis by Colmers et al. found an increased breast cancer risk in glargine users compared to those who did not use glargine [33]. This may be attributed to the mitogenic and proliferative activity of glargine, due to its increased binding affinity to insulin-like growth factor-1 compared to human insulin [34]. However, a pooled analysis of 13 epidemiological studies did not support the association between treatment with glargine and increased incidence of breast cancer [34].

There are preclinical data supporting the antineoplastic effect of statins. Possible mechanisms include reduced expression of the antiapoptotic protein bcl-xL and increased transcription of phosphatase and tensin homolog, which inhibit the oncogenic phosphatidylinositol-3-kinase pathway [35, 36]. Several* in vitro* studies demonstrated that statins decrease breast cancer cell proliferation [35, 37–39]. A recent meta-analysis by Zhong et al. found that statins usage was associated with improved cancer survival [40]. Despite these reports, current data do not support lower incidence of breast cancer in patients treated with statins [41–43] and it is not clear if they have any effect on outcome [44, 45].

Thyroid function is an additional metabolic factor that may influence breast cancer. Data regarding a correlation between higher levels of free T4 (FT4) and triiodothyronine, as well as lower levels of thyroid peroxidase autoantibodies (TPO-Ab) and higher breast cancer incidence, are accumulating [46–49]. Some data suggest that FT4 and TPO-Ab levels are associated with breast cancer characteristics [48]; however, the impact of levothyroxine treatment is unclear.

The aim of this study was to evaluate the association of metformin, insulin, statins, and levothyroxine and breast cancer characteristics and outcome. With the advent of ODX as a prognostic and predictive tool for daily practice, important molecular information on tumor in addition to traditional pathology report is obtained [9, 10]. Therefore, we chose to focus on patients with early breast, ER positive, HER2 negative disease, for whom their physician added the ODX test prior to making a decision on systemic treatment.

## 2. Methods

This was a retrospective single center cohort study. Patients with early ER positive, HER2 negative breast cancer, who were diagnosed between 4/2005 and 3/2012 were included. The cohort included all patients treated in our institute during this period whose tumors were sent for ODX analysis.

The medical records of all patients were retrospectively reviewed up to 8/2015. Data on patient demographics, clinical-pathological parameters, treatment, and outcome were retrieved. Data regarding usage of metformin, insulin, statins, and levothyroxine at the time of breast cancer diagnosis were also collected. Duration of treatment until breast cancer diagnosis and related comorbidities including DM, dyslipidemia, and hypothyroidism were retrieved from the Clalit Health Service database. We evaluated the influence of the studied medications on tumor size, nodal involvement, stage, ODX recurrence score (RS), and histological characteristics. Histological characteristics evaluated were ER, PR, HER2, grade, angiolymphatic and perineural invasion, Ki67, P53, and histological subtype. As angiotensin converting enzyme inhibitors (ACEI) and angiotensin receptor blockers (ARB) are commonly prescribed in patients with DM and several studies suggested a possible relationship between these medications and breast cancer [50, 51], we evaluated their usage in this cohort. The study protocol was approved by the institutional ethics committee.

Staining for ER, PR, p53, Ki-67, and HER2 was performed using the Ventana Benchmark XT automated immunostainer (Ventana, Tucson, AZ, USA) with the standard cell conditioner (CC1) protocol for 30 min. Following deparaffinization and the CC1 protocol, ready-to-use ER rabbit monoclonal antibody [anti-ER (6F11) primary antibody; Ventana] was applied for 40 min incubation at 37°C; PR rabbit monoclonal antibody (clone 16; Novocastra, Newcastle, UK) was employed at a 1 : 100 dilution with 40 min incubation at 37°C; Ki 67 rabbit monoclonal antibody (clone SP6; Thermo Fisher Scientific) was used at a 1 : 100 dilution for 40 min at 37°C; and ready-to-use PATHWAY HER2 anti-HER2/neu rabbit monoclonal antibody (4B5) (Ventana) was utilized with 32 min incubation at 37°C. Slides were counterstained with 4′,6-diamidino-2-phenylindole (Sigma-Aldrich), and the stained material was visualized under a BX51 fluorescence microscope (Olympus). The signals were analyzed manually.

ER and PR immunohistochemical (IHC) staining used the modified version of the *H*-score method, 1 × percentage of weakly staining nuclei + 2 × percentage of moderately staining nuclei + 3 × percentage of intensely staining nuclei/100, yielding a range of 0 to 3. Ki67 staining was reported by percentage of positively stained nuclei (0 to 100%). HER2 negativity was defined as an immunohistochemistry test score of 0 or 1. If the IHC score equaled 2, HER2 negativity was determined according to fluorescence* in situ* hybridization test per American Society of Clinical Oncology guidelines at the time of the test.

## 3. Statistical Analysis

Statistical analysis was performed using SAS Software, version 9.4. *t*-test was used to compare the value of continuous variables between study groups. Chi-square (for more than two groups) or Fisher's exact test (for two groups) were used to compare the value of categorical variables between study groups. Pearson correlation was used to assess the associations between continuous variables. Continuous variables were presented by mean ± standard deviation (SD). ANOVA test was used to assess associations between categorical variables. Categorical variables were presented by (*N*, %). In order to evaluate the association between medications usage duration and the characteristics that were found significant, we used the duration of usage as a continuous variable.

Overall survival (OS) was assessed by Kaplan-Meier survival analysis, with the log-rank test. Breast cancer specific survival (BCSS) and disease-free survival (DFS) were assessed by the Cox proportional hazards model, with the Fine and Gray correction for noncancer death as a competing risk. Two-sided *p* values less than 0.05 were considered statistically significant. To evaluate interaction between metformin and insulin we performed multivariate analysis for tumor characteristics which were found statistically significant on univariate analyses. We added interaction of metformin and insulin to the model; *p* value less than 0.15 was considered to be significant for interaction.

## 4. Results

### 4.1. Patients and Tumor Characteristics

A total of 671 patients were included in the study. Median age was 61 years (range 34–85). There were 662 women and 9 men. Among the women, 446 (67.4%) were postmenopausal. History of invasive and noninvasive breast cancer was documented in 11.2% and 1.9% of the patients, respectively. Nine percent of the patients had a history of malignancy of nonbreast origin. Personal history of benign breast disease was noted in 9.1%. First and second degree family history of breast cancer or any other cancer were noted in 39.7% and 58%, respectively. Breast cancer diagnosis was established by screening in the majority of cohort population (82.6%). Screening rates were similar among the studied groups.

Data regarding patient comorbidities were missing for 160 (23.8%) patients. History of DM was documented in 88 (17.2%), dyslipidemia in 333 (65.2%), hypothyroidism in 74 (14.5%), and hyperthyroidism is 6 (1.2%) patients. For 177 (26.4%) patients data regarding duration of medications usage were not available. Data regarding medication usage were missing for 15 patients.

Tumor characteristics for the entire population cohort and for each medication group are detailed in Tables 1 and 2. As expected, there were correlations between the intensity of ER and PR IHC staining and the Oncotype ER, PR, and HER2 (according to RT-PCR results, *p* < 0.0001 for all variables). Median ODX RS was 17 (range 0–88) and 50.4%, 38.4%, and 11.2% of the patients had low (0–17), intermediate (18–30), and high (≥31) RS, respectively.

### 4.2. Impact of Diabetes Mellitus and Related Medications on Tumor Characteristics

**Figure 1:**
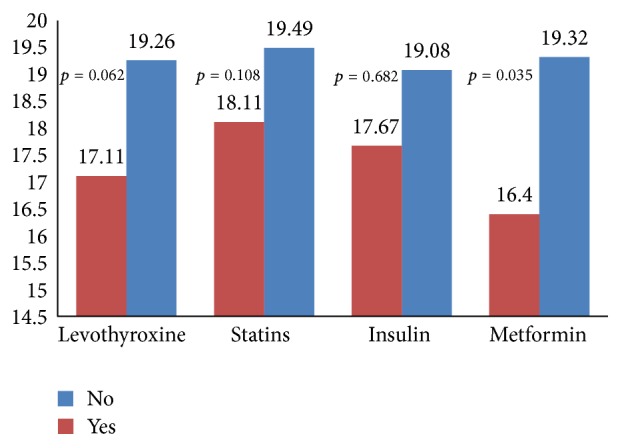
Oncotype DX recurrence score according to medication usage.

Patients with DM had lower ODX RS (mean 16.7 ± 10 versus 19.7 ± 20.7, *p* = 0.014). Tumor size, nodal involvement, and other histological characteristics were not influenced by diagnosis of DM. At breast cancer diagnosis, 60 (9.1%) patients were treated with metformin. Median duration usage was 50.5 (1–80.5) months and interquartile range (IQR) was 22–80.5 months. Metformin usage was associated with lower ODX RS (mean 16.4 ± 8.5 versus 19.3 ± 10.4, *p* = 0.035) (Figure 1) and more intense ER staining (mean 2.59 ± 0.4 versus 2.46 ± 0.58, *p* = 0.032). These differences were not associated with the duration of metformin treatment. Other histological characteristics, as well as tumor size, nodal involvement, and stage, were comparable. Nine (1.4%) patients were treated with insulin at breast cancer diagnosis. Median duration of insulin usage was 56 (10–126) months and IQR was 15–94 months. Angiolymphatic invasion was more common among these patients (22.2% versus 5.8%, *p* = 0.041), while the Ki67 percentage was lower (mean 9.5% ± 4.64 versus 16% ± 13.64, *p* = 0.017). Duration of insulin treatment did not influence these findings. Insulin usage was not associated with ODX RS, tumor size, nodal involvement, and other histological characteristics. For ODX RS, ER staining, and angiolymphatic invasion rate there were no significant interactions between metformin and insulin in the multivariate analysis (*p* = 0.199, *p* = 0.413, and *p* = 0.989, resp.). Treatment with either ACEI or ARB was documented in 38 (63.3%) patients treated with metformin and in 6 (66.7%) patients treated with insulin. Neither ARB nor ACEI were associated with ODX RS, intensity of ER stain, or angiolymphatic invasion rate.

### 4.3. Impact of Dyslipidemia and Statins on Tumor Characteristics

Treatment with statins was noted in 208 (31.7%) patients with median duration usage of 72 (1–168) and IQR of 39–108 months. Neither dyslipidemia nor treatment with statins was associated with the tumor characteristics examined.

### 4.4. Impact of Thyroid Dysfunction and Levothyroxine on Tumor Characteristics

Hyperthyroidism and hypothyroidism did not influence all the evaluated characteristics. Sixty-two (9.4%) patients were treated with levothyroxine. Median duration usage was 113 (8–168) months and IQR was 85–130 months. These patients had a trend toward lower ODX RS (mean 17.1 ± 8.35 and 19.26 ± 10.43, *p* = 0.062) (Figure 1). Prolonged levothyroxine treatment was associated with lower ODX RS (*p* = 0.005). Histological subtype distribution differed. IDC, ILC, and other histologies were found in 69.4%, 16.1%, and 14.5%, respectively, for patients treated with levothyroxine compared to 82.1%, 11.8%, and 6.1%, respectively, for patients not treated with levothyroxine (*p* = 0.02). Duration of treatment had no significant impact on this histological difference. Levothyroxine treatment was not associated with other tumor characteristics.

### 4.5. Treatment

Adjuvant hormonal treatment was prescribed to 97.7% of the patients. Most patients (74.4%) did not receive adjuvant chemotherapy. Patients treated with levothyroxine were less likely to receive adjuvant chemotherapy (6.4% versus 27.2%, *p* = 0.0001). For patients treated with metformin, there was a trend toward receiving adjuvant chemotherapy less often (15.5% versus 26.2%, *p* = 0.082). Other evaluated medications have not been associated with chemotherapy and hormonal therapy usage rates.

### 4.6. Outcome

**Figure 2:**
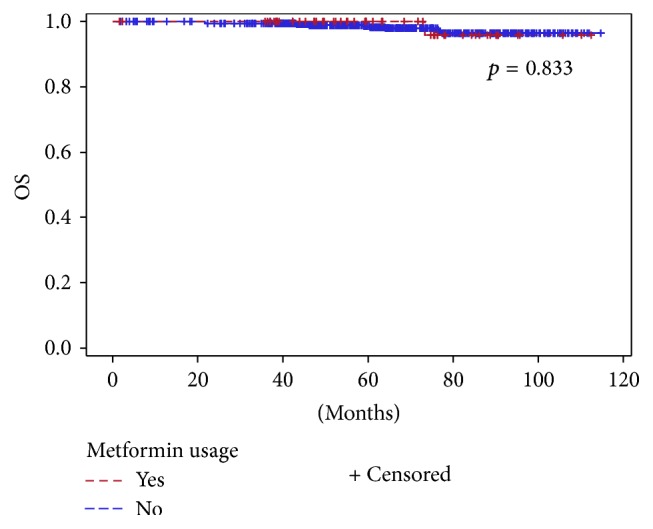
Overall survival according to metformin usage.

**Figure 3:**
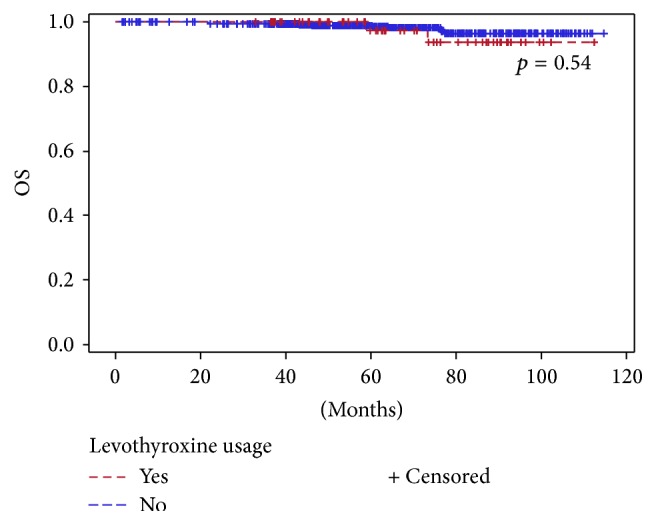
Overall survival according to levothyroxine usage.

Median follow-up was 61.8 months (range 1.7–114.6). During this period, 13 (1.9%) patients died of breast cancer, 644 (95.9%) remained alive, and 14 (2.1%) died of other causes. The estimated 5-year DFS rates for all patients were 95.7%, with rates of 96.7%, 95.7%, and 91.9%, for patients with low, intermediate, and high ODX RS, respectively. Five-year DFS rates were significantly different among patients with low to high RS (HR = 0.4, 95% CI: 0.16–0.99, *p* = 0.047). Five-year OS rate for the whole population was 98.5%. The Cox proportional hazards model was not applicable for patients treated with insulin due to the small number of patients. Five-year DFS, BCSS, and OS rates were comparable in the studied medications groups and related comorbidities. Figures 2 and 3 depict OS according to metformin and levothyroxine usage, respectively.

## 5. Discussion

This study evaluated the association between four commonly used drugs, metformin, insulin, statins, and levothyroxine, and breast cancer. The first three are frequently used in patients with metabolic syndrome. In an attempt to better understand the direct effects of these medications, the influence of the related comorbidities was also evaluated. Data analysis revealed several significant findings. Patients with diabetes as well as patients treated with metformin had a significantly lower ODX RS. In addition, metformin treatment was associated with more intense ER staining. To the best of our knowledge, these findings are novel. They concur with previous reports describing improved survival in breast cancer patients treated with metformin [26, 29]. Nonetheless, as the majority of the patients with DM were treated with metformin, it is not clear whether the difference in ODX RS relates to direct effect of metformin or the existence of DM.

The significantly lower ODX RS in patients treated with metformin did not translate into improved outcome in our cohort. Given the excellent outcome for all the subgroups, a larger population and longer follow-up are probably required to identify such differences. The tendency of patients treated with metformin to receive less adjuvant chemotherapy treatment is consistent with lower ODX RS.

Patients treated with insulin had significantly higher angiolymphatic invasion in their tumors; however, the Ki67 percentage was significantly lower. While evidence of higher angiolymphatic invasion could be consistent with previous reports implying exogenous insulin as a growth factor for cancer cells [34], lower Ki67 is inconsistent with this postulation. Of note, since only nine patients in the cohort population were treated with insulin, the conclusions that can be drawn from this specific analysis are limited. Nonetheless, as large scaled epidemiological studies imply existing association between insulin usage and breast cancer incidence and mortality [52, 53], further research to evaluate the potential effect of insulin on breast cancer is needed.

As opposed to the antidiabetic drugs, which might suggest some impact on breast cancer biology, we did not detect any association between breast cancer and statins treatment. This is in contrast to the favorable effect described by some investigators [35, 36, 40]. The findings are confined to ER positive, HER2 negative, early breast cancer patients and it is possible that statins could affect other breast cancer subgroups.

The fourth drug investigated was levothyroxine. While neither diagnosis of hypothyroidism nor diagnosis of hyperthyroidism had influence on tumor characteristics, patients treated with levothyroxine were less likely to have IDC subtype and had a tendency to have a lower ODX RS and extended levothyroxine treatment was associated with lower ODX RS. These results suggest that levothyroxine might have a role in breast cancer pathophysiology. Despite the association with extended levothyroxine usage and lower ODX RS, levothyroxine usage was not associated with improved outcome. As all patients had excellent survival, longer follow-up is probably needed to evaluated differences in outcome. To the best of our knowledge, all of the aforementioned findings have not been described previously.

Our study has several limitations. It was a retrospective study, which may cause bias due to unknown or unrecorded confounders. Patients' comorbidities might cause potential confounders and add to the difficulty of results interpretation, as was previously described in another study [16]. As this was a single center study, it is more vulnerable to unknown bias. Data regarding comorbidities and duration of medications usage were retrieved from the largest health service organization in Israel. As not all patients are affiliated to this organization, these data were missing for a quarter of the patients. Information regarding other antidiabetic drugs, which might have some influence on breast cancer, was not collected. Moreover, the efficacy of the evaluated medications, including hemoglobin A1C%, cholesterol level, and thyroid function were not documented. These data might help us to better understand the influence of the evaluated medications on breast cancer. The study included all patients with ER positive, HER2 negative, early breast cancer, from both genders. Male breast cancer might represent a distinct clinical entity; therefore their inclusion might have caused additional bias. Nonetheless, as only 9 (1.3%) men were included in the study cohort, they were not likely to significantly influence our results.

Strengths of this study include the large patient cohort. Furthermore, the chart review included detailed patient data, which is lacking in registry-based studies. The correlation between IHC staining results and the ODX ER, PR, and HER2 (based on RT-PCR) results, as well as the correlation between ODX RS and the DFS of the study population, add to the reliability and validity of the findings.

## 6. Conclusion

Patients with ER positive, HER2 negative, early breast cancer who were treated with metformin had lower ODX RS and more intense ER staining, which are both associated with favorable outcome. This supports recent research efforts to incorporate metformin in anticancer adjuvant treatment. Patients treated with levothyroxine also had distinct tumor characteristics. Our findings might suggest that these medications have a role in the pathophysiology and development of breast cancer. As they are commonly prescribed, additional studies are required to elucidate the possible associations between these medications and breast cancer and to explore possible clinical implications.

## Figures and Tables

**Table 1:** Tumor burden and Oncotype DX recurrence score.

Population (*N*)	*T*		Macroscopic node positive^1^		Stage (%)				Oncotype Dx RS	
	Mean size cm (SD)	*p* ^2^	(%)	*p* ^2^	I	II	III	*p* ^2^	Mean (SD)	*p* ^2^
All (671)	1.67 (0.79)	—	9	—	71.2	28.3	0.5	—	19.06 (10.27)	—
Metformin^3^ (60)	1.72 (0.79)	0.621	13.3	0.217	65	35	0	0.89	16.4 (8.52)	0.035
Insulin^3^ (9)	2.03 (0.6)	0.171	11.1	0.574	44.4	55.6	0	0.727	17.67 (13.64)	0.682
Statins^3^ (208)	1.77 (0.95)	0.067	9.2	0.994	67	32.5	0.5	0.226	18.11 (10.3)	0.108
Levothyroxine^3^ (62)	1.61 (0.68)	0.509	6.5	0.464	69.4	30.6	0	0.949	17.1 (8.35)	0.062

^1^Macroscopic nodes-lymph node metastases > 2 millimeter.

^2^ *p* value refers to comparison of each variable between patients who used the specified medication to the rest of the cohort.

Medication usage was not available for 15 patients.

RS: recurrence score.

^3^Median duration of medications usage (months, range): metformin 50.5 (1–80.5), insulin 56 (10–126), statins 72 (1–168), and levothyroxine 113 (8–168).

**Table 2:** Histological characteristics.

Population *(N)*	Histology				Grade		ER		PR		Ki67 (%)		P53 (%)		Angiolymphatic invasion		PNI	
	IDC (%)	ILC (%)	Other^2^ (%)	*p* ^1^	Mean (SD)	*p* ^1^	Mean (SD)	*p* ^1^	Mean (SD)	*p* ^1^	Mean (SD)	*p* ^1^	Mean (SD)	*p* ^1^	Yes (%)	*p* ^1^	Yes (%)	*p* ^1^
All (671)	80.9	12.2	6.9	—	2.02 (0.59)	—	2.47 (0.57)	—	1.47 (1.19)	—	15.91 (13.58)	—	6.45 (16.96)	—	6.1	—	4.5	—
Metformin^3^ (60)	76.7	15	8.3	0.683	2 (0.63)	0.846	2.59 (0.4)	0.032	1.29 (1.05)	0.217	13.7 (11.1)	0.204	6.96 (14.7)	0.828	3.4	0.368	5.1	0.742
Insulin^3^ (9)	77.8	11.1	11.1	0.878	1.86 (0.38)	0.474	2.49 (0.67)	0.948	1.12 (0.94)	0.371	9.5 (4.64)	0.017	8.5 (20.3)	0.765	22.2	0.041	0	0.512
Statins^3^ (208)	79.8	11.1	9.1	0.266	2.07 (0.5)	0.193	2.48 (0.55)	0.916	1.41 (1.02)	0.346	16.78 (14.53)	0.318	6.45 (15.61)	0.999	7.8	0.212	4.9	0.768
Levothyroxine^3^ (62)	69.4	16.1	14.5	0.02	2.02 (0.55)	0.94	2.47 (0.52)	0.999	1.37 (1.27)	0.488	15.28 (12.64)	0.727	5.92 (16.56)	0.84	3.3	0.339	1.6	0.257

^1^ *p* value refers to the comparison of each variable between patients who used the specified medication to the rest of the cohort.

^2^Another subtype histology includes medullary, mucinous, papillary, and tubular carcinomas.

Medication usage was not available for 15 patients.

ER: estrogen receptor, PNI: perineural invasion, and PR: progesterone receptor.

^3^Median duration of medications usage (months, range): metformin 50.5 (1–80.5), insulin 56 (10–126), statins 72 (1–168), and levothyroxine 113 (8–168).
